# Air humidity and annual oscillations in ^90^Sr/^90^Y and ^60^Co decay rate measurements

**DOI:** 10.1038/s41598-022-13841-7

**Published:** 2022-06-09

**Authors:** S. Pommé, K. Pelczar, I. Kajan

**Affiliations:** 1 European Commission, Joint Research Centre (JRC), Geel, Belgium; 2 Paul Scherrer Institut (PSI), Villigen, Switzerland

**Keywords:** Physics, Space physics, Solar physics, Astronomy and astrophysics

## Abstract

Parkhomov published decay rate measurements of ^90^Sr/^90^Y and ^60^Co beta decay sources with Geiger–Müller counters which showed annual cyclic deviations with less than 0.2% amplitude from a purely exponential slope. He investigated instrument instability induced by environmental parameters, yet did not find a clear coincidence with local temperature, atmospheric pressure, and relative humidity. Parkhomov hypothesised that gravitationally-focussed ‘slow’ cosmic neutrinos influenced beta decay. In the current work, environmental conditions in the Moscow area at the time of the experiment are presented. There appears to be a resemblance of the shape of the annual ^90^Sr/^90^Y decay rate anomalies with the inverse of the absolute air humidity, albeit with an apparent time shift of 0.05–0.15 year. Humidity may have influenced the range of beta particles in air, as well as geometric and electronic properties of the detection set-up, however causality could not be unambiguously demonstrated. The instabilities in the ^60^Co data were more difficult to correlate with environmental data, except for some similarities with temperature and external dew point.

## Introduction

In recent years, there has been a lively discussion on the origin of observed anomalies in some time series of measured decay rates^1^. The most notable cases in which the effect occurred pertained to counting of beta emissions with Geiger–Müller (G–M) counters^2–6^ or set-ups with unstable conditions^7, 8^, whereas it is mostly negligible in measurements with the accurate techniques^9–13^ of primary standardisation laboratories^14–23^ and other laboratories working under stable measurements conditions^24–29^. Parkhomov^5^ published decay rate measurements of ^90^Sr/^90^Y and ^60^Co beta decay sources with G–M counters and observed annual cyclic perturbations with less than 0.2% amplitude. There is no reason to associate these fluctuations with anomalies in the decay process itself; Accurate activity measurements of ^90^Sr/^90^Y with the TDCR technique (Triple-to-Double Coincidence Rate counting^12^) are free of any annual oscillation exceeding 0.004% amplitude^15, 16, 18^. Ionisation chamber measurements^11^ of ^60^Co sources are free of annual oscillations at an accuracy level of 0.007%^16, 18^.

The most likely explanation is found in environmental conditions that influence the electronic operation and geometric conditions of the experiment. Recent studies have revealed that ambient air humidity can significantly influence decay rate measurements, even inside temperature-controlled laboratories. Notable examples are time series of ^32^S beta decay at Brookhaven^2, 30^, ^32^P beta decay at Ohio State University^4, 30^, radon decay in a closed canister at the Geological Survey of Israel^9, 31–33^, various beta emitters at the Technical University of Valencia^6, 34, 35^, and γ-ray spectrometry measurements of ^145^Sm and ^171^Tm at the Paul Scherrer Institute^36, 37^. The perturbations from a smooth exponential decay curve revealed strong correlations with humidity represented by the dew point, a humidity accumulation model, or absolute air humidity derived from historic data of nearby weather stations.

In spite of this straightforward explanation of instrumental instability, some authors have questioned the invariability of the decay constants and made assertions that a significant fraction of radioactive decay is induced by solar and cosmic neutrinos^1^. Metrologists have often argued that there is no metrological ground for these assertions, since the best measurements point to the validity of the exponential-decay law^14–39^. Parkhomov hypothesised that gravitationally-focussed ‘slow’ cosmic neutrinos influenced beta decay. In this work, environmental conditions in the Moscow area at the time of the experiment are presented. Particular attention is paid to the annual cycles of absolute air humidity, which may have influenced the experiment.

### The ^90^Sr/^90^Y case

**Figure 1:**
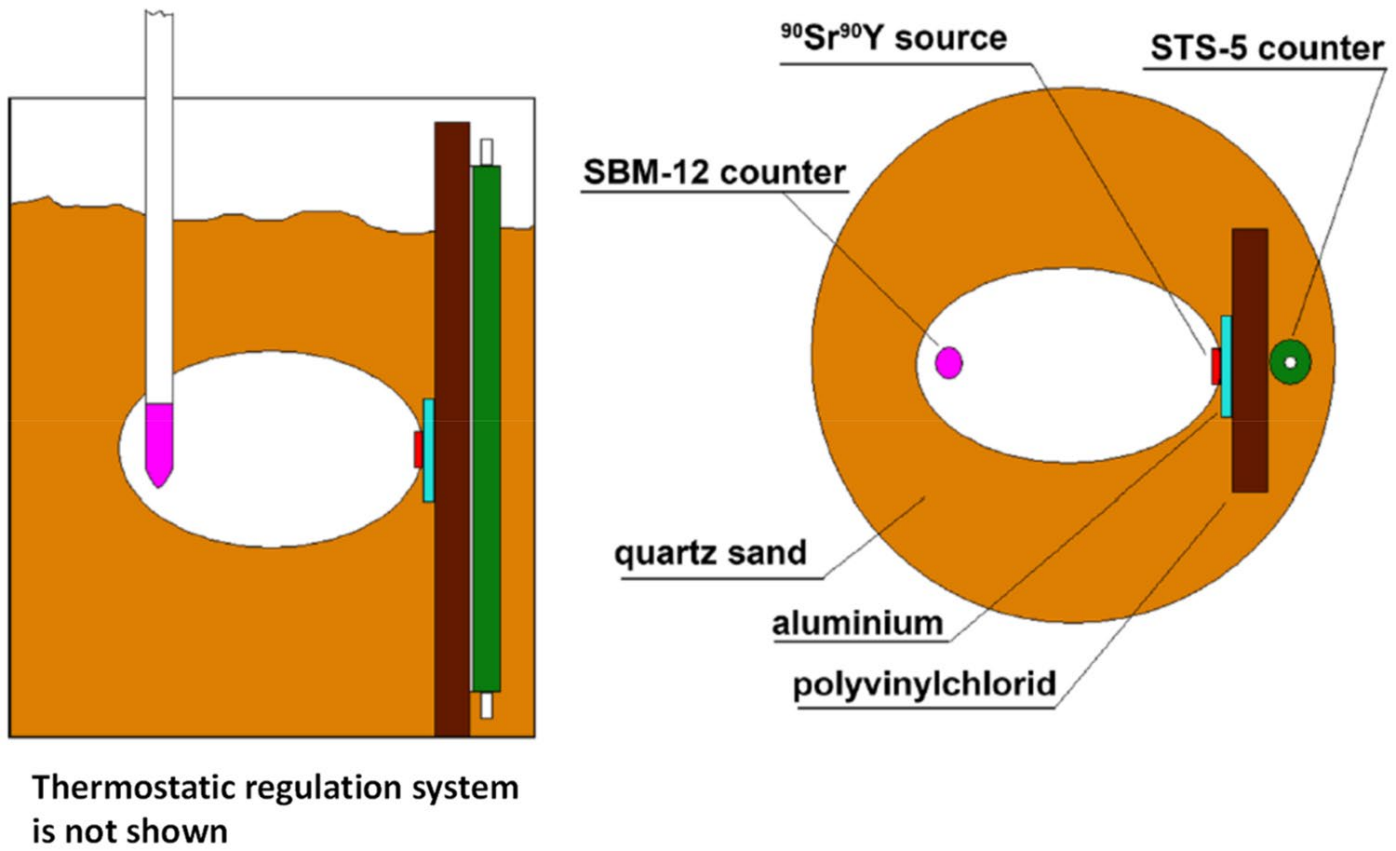
Scheme of the sand-filled cylindrical container with the ^90^Sr/^90^Y source and the SBM-12 and STS-5 Geiger counters used by Parkhomov^5^.

At the Russian Academy of Natural Sciences in Moscow (Russia), Parkhomov built a set-up to investigate rhythms and fluctuations in radioactivity and various processes (low-frequency noise in semiconductor devices, oscillations of quartz resonators), and simultaneously recorded temperature and radiation background. Of particular interest are the measurements of ^90^Sr/^90^Y beta decay rates with the SBM-12 and STS-5 Geiger-Müller counters presented in Fig. 1. The ^90^Sr/^90^Y parent-progeny pair is in equilibrium, with ^90^S emitting a soft beta spectrum with 546 keV endpoint energy and ^90^Y a harder beta spectrum up to 2.3 MeV. Both beta spectra are detected in the first counter, type SBM-12, located in the air cavity at a distance of 2 cm from the source. The second counter, type STS-5; is separated from the source by additional layers of aluminium and polyvinylchloride, thus recording mainly the high-energy fraction of the ^90^Y beta spectrum. The temperature in the installation is stabilised at 31 °C ± 0.1 °C. The container with source and detectors is filled with quartz sand to absorb externally emitted beta particles.

In private communication, Parkhomov has kindly provided measurement data in an effort to reproduce the departures from a smooth exponential decay curve, resulting in three data sets:

1. An extensive set of intermediately processed data that were the basis of the final data prior to further filtering and correcting.
2. A set of processed data, corrected for decay and change in response of the detector with time, averaged over 31–38 h (SBM-12) or 38–48 h (STS-5), and excluding data for which the thermostat indicated less than 20 °C or more than 31 °C.
3. Visually matching analogues of the original graphs in Parkhomov’s publications^5^. These graphs are similar but not identical to data set #2.

**Figure 2:**
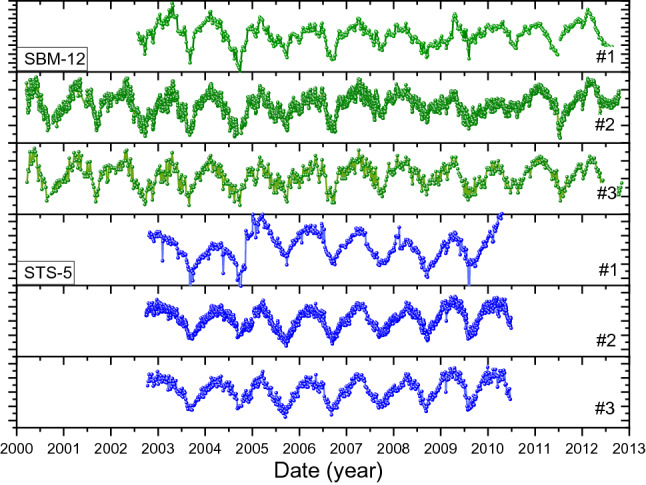
Versions #1, #2, #3 of the decay-corrected count rates from ^90^Sr/^90^Y beta decay detected in the SBM-12 and STS-5 counters. The #1 data were averaged over evenly distributed time intervals. The #2 set was a reanalysis provided by Parkhomov, whereas #3 is a visual analogon of previously published graphs^5^.

The three versions of the SBM-12 and STS-5 data sets are compared in Fig. 2. The intermediately processed data set #1 shows anomalies and the annual cycle is partly dissimulated. The filtered data set #2 shows a smoother annual cycle, albeit that the published data set #3 is more regular. According to Parkhomov, the service life of the counters was exhausted at the end of their respective measurement periods. This resulted in abrupt changes in the counting rate of the order of 0.1% and a higher chance for the appearance of false pulses. The thermostat was unable to maintain the reference temperature when the room temperature exceeded the range 20–31 °C, thus necessitating the elimination of the corresponding measurement data. These instabilities were partially corrected for in datasets #2 and #3. The data obtained after January 1, 2010 for SBM-12 and after July 1, 2009 for STS-5 should be considered as indicative.

**Figure 3:**
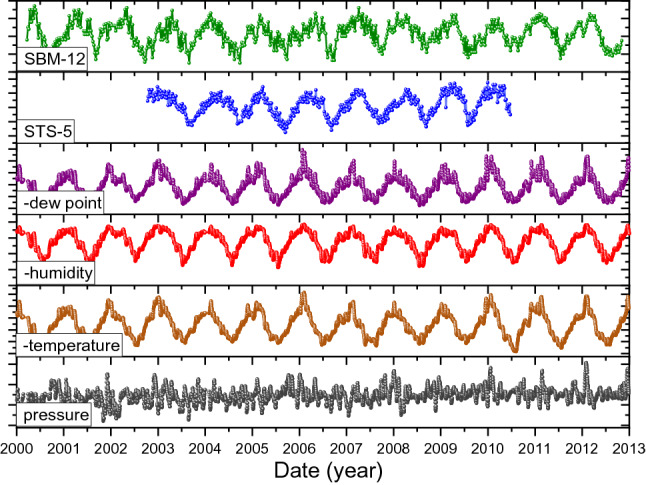
The shape of the variations in the ^90^Sr/^90^Y decay rates measured with the SBM-12 and STS-5 G–M counters by Parkhomov^5^, compared to historical weather data in Moscow^35^. The dew point, humidity and temperature data are presented in reverse scale for direct comparison with the decay measurements.

The data sets #3 are shown in Fig. 3, together with historical weather variables extracted from the weather station of Sheremetyevo international airport in Moscow^40^. Even though the laboratory is at the other side of Moscow, it can be assumed that the general trends of the weather conditions run largely in parallel. The graph shows the dew point, the absolute humidity (calculated with a heuristic formula from temperature and relative humidity), temperature in reverse scale, as well as the ambient air pressure in normal scale. It appears that the shape of the oscillations in the air humidity shows some resemblance to the instabilities in the ^90^Sr/^90^Y decay rate measurement results.

## Interpretation of ^90^Sr/^90^Y data

**Figure 4:**
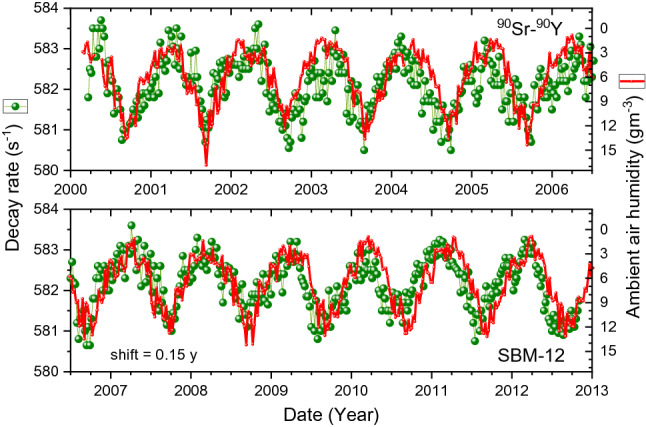
Decay-corrected count rates of ^90^Sr/^90^Y beta decay obtained with the SBM-12 G–M counter (dots), compared to absolute humidity in Moscow calculated from weather data shifted in time by 0.15 year to the right (line).

**Figure 5:**
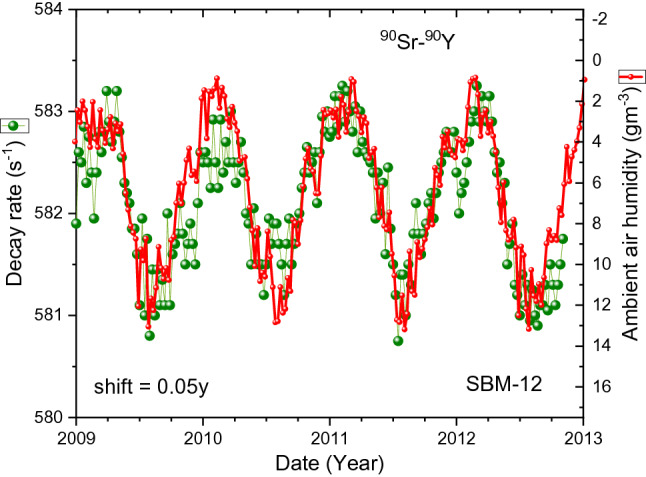
Decay-corrected count rates of ^90^Sr/^90^Y beta decay obtained with the SBM-12 G–M counter (dots) from 2009 to 2013, compared to absolute humidity in Moscow calculated from weather data shifted in time by 0.05 year to the right (line).

**Figure 6:**
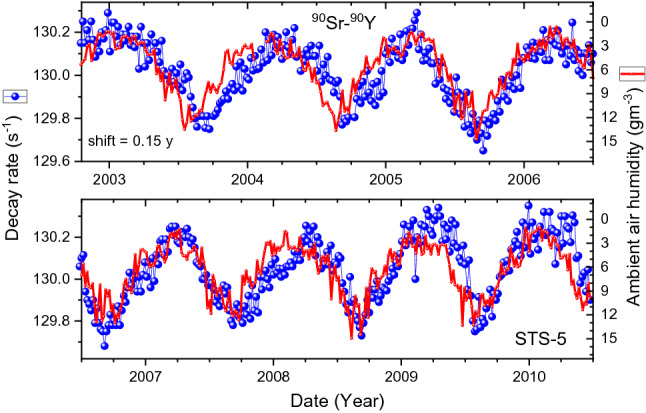
Decay-corrected count rates of ^90^Sr/^90^Y beta decay obtained with the STS-5 G–M counter (dots), compared to absolute humidity in Moscow calculated from weather data shifted in time by 0.15 year to the right (line).

**Figure 7:**
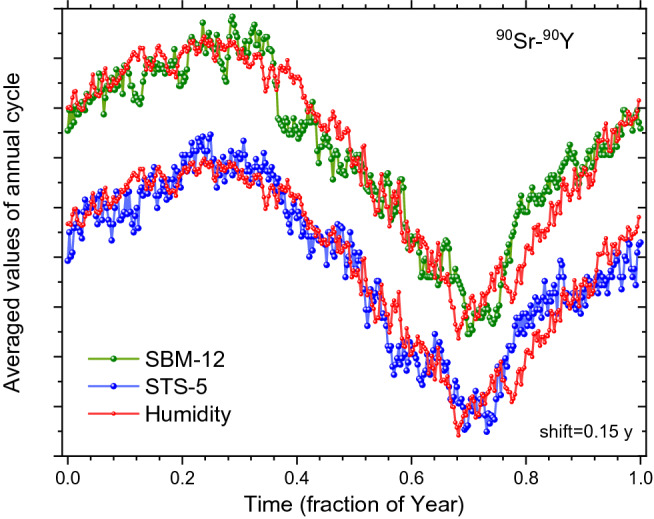
Averaged decay-corrected count rates of ^90^Sr/^90^Y beta decay obtained with the SBM-12 (top) and STS-5 G–M counter (bottom) as a function of time in the year, compared to absolute humidity in Moscow calculated from weather data shifted in time by 0.15 year to the right (line).

In Fig. 4, an overlay is shown of the SBM-12 data set #3 with the absolute humidity data. There appears to be a resemblance of the shape of the annual decay rate anomalies with the absolute air humidity (in reverse scale), albeit with an apparent time shift of 0.15 year. The time shift appears to be smaller, about 0.05 year, during the period from 2009 to 2013, as demonstrated in Fig. 5. To a large extent, also the STS-5 data in Fig. 6 resemble the variations in humidity with the same delay of 0.15 year. Moreover, when the data are averaged over the time of the year in Fig. 7, one finds a rather good match between the shapes over the major part of the year.

Humidity may have influenced the range of beta particles in air, as well as geometric and electronic properties of the detection set-up. Since the beta particles enter the detector with a continuous energy spectrum, the threshold level separates the detected from the non-detected fraction. Any change in the threshold energy, the amplification of the gas detector, or the energy spectrum of the electrons (after straggling in air with a variable density) will result in a different effective detection efficiency. It is interesting to note that Parkhomov does not consider any variability in the threshold region and assumes that variations merely occur at the high-energy side resulting from inverse beta decay.

It is tempting to ascribe the variations in the decay rates to the influence of humidity, however the consideration that the effect of the humidity appears to be delayed by about 55 days puts this hypothesis under pressure. It is conceivable that two environmental conditions have executed a combined effect on the set-up. A weighted combination of temperature and humidity can result in waves that are delayed in time.

## The ^60^Co case

The ^60^Co decay rate measurements were performed with a miniature (diameter 2 mm, length 6 mm) halogen SBM-12 G–M counter. A 0.1-mm-thick kovar alloy (nickel, cobalt and iron) tube was tightly clad to the counter. The ^60^Co was formed by irradiation of this alloy in a nuclear reactor. The ^60^Co decay counting rate during the measurement campaign decreased from 16 to 5 s^−1^, whereas the background counting rate was about 0.01 s^−1^. For statistical precision, the data were averaged over long periods, from 2.5 days at the beginning to 8 days at the end. The SBM-12 detector was not thermally stabilised in view of the assumed weak dependence of the counting rate on temperature (0.0006% per °C).

**Figure 8:**
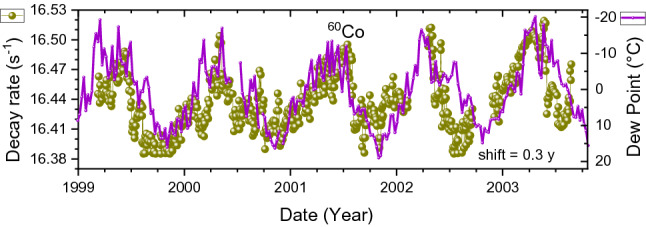
Averaged decay-corrected count rates of ^60^Co beta decay obtained with an SBM-12 G–M counter (dots) as a function of time, compared to the dew point in Moscow shifted in time by 0.3 year to the right (line).

The ^60^Co decay rates show annual oscillations that look more acute than a sinusoidal function, as shown in Fig. 8. There is no convincing match with the absolute air humidity, even though one would suspect a similar sensitivity to humidity of the SBM-12 G–M counter as for ^90^Sr/^90^Y. However, the ^60^Co source was not surrounded by sand, therefore humidity was not buffered by a surrounding medium. The shape of the oscillations resembles the time dependency of the dew point, albeit with an unrealistic time shift of 0.3 year. In spite of the similarity of both time series, the large time gap suggests that the similitude may be merely fortuitous.

**Figure 9:**
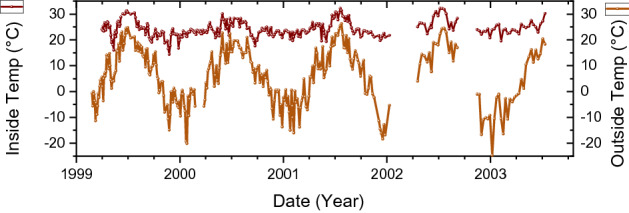
Comparison of daily average outside temperature from a weather station in Moscow^40^ with inside temperature in the laboratory measured by Parkhomov. The inside temperature is more stable, yet also rises above average during the summer peaks.

**Figure 10:**
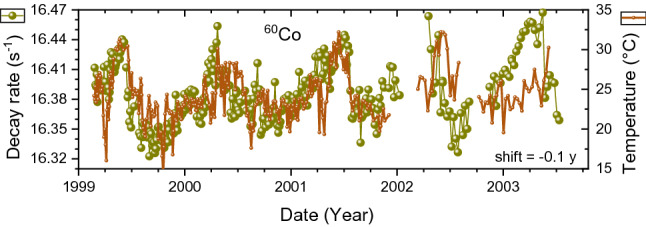
Averaged decay-corrected count rates of ^60^Co beta decay obtained with an SBM-12 G–M counter (dots) as a function of time, compared to the inside temperature shifted in time by 0.1 year to the left (line).

The reality may be more complex and it is difficult to disentangle the effects of environmental factors retrospectively. The decay rates were not exclusively influenced by outside weather conditions, since the laboratory was heated in the cold season (from September to May), electric heaters were used during cold snaps, and air conditioners during hot weather. The difference between outside temperature from the weather station and the temperature measured inside the laboratory by Parkhomov is shown in Fig. 9. For illustration purposes, in Fig. 10 the ^60^Co count rates are compared with the temperature inside the laboratory shifted by 0.1 year to left. There is a vague resemblance in the time region 1999–2002, however there is no satisfactory evidence for causality.

## Conclusions

The annual variations in the measured ^90^Sr/^90^Y beta decay rates show a marked resemblance with the annual variations in the absolute air humidity in the Moscow region, except that the humidity cycle precedes the decay rate cycle by about 0.05–0.15 year. The ^90^Sr/^90^Y source and counters were surrounded by sand, which may have acted as a buffer for humidity, which in turn may have influenced the range of beta particles in air and capacitance in electronics. The ^60^Co decay rates were measured in other conditions and were difficult to interpret. The variations may have been caused by a combination of environmental parameters, e.g. humidity and temperature, as well as artificial heating and air conditioning inside the laboratory.

## Data Availability

The datasets used and/or analysed during the current study available from the corresponding author on reasonable request.
